# Human Vδ2^+^ γδ T Cells Differentially Induce Maturation, Cytokine Production, and Alloreactive T Cell Stimulation by Dendritic Cells and B Cells

**DOI:** 10.3389/fimmu.2014.00650

**Published:** 2014-12-19

**Authors:** Andreea Petrasca, Derek G. Doherty

**Affiliations:** ^1^Division of Immunology, School of Medicine, Trinity College Dublin, University of Dublin, Dublin, Ireland

**Keywords:** human γδ T cells, dendritic cells, B cells, cytokines, antibody production, APC, T cell proliferation

## Abstract

Human γδ T cells expressing the Vγ9Vδ2 T cell receptor can induce maturation of dendritic cells (DC) into antigen-presenting cells (APC) and B cells into antibody-secreting plasma cells. Since B cells are capable of presenting antigens to T cells, we investigated if Vγ9Vδ2 T cells can influence antigen-presentation by these cells. We report that Vγ9Vδ2 T cells induced expression of CD86, HLA-DR, and CD40 by B cells and stimulated the release of IL-4, IL-6, TNF-α, and IgG, IgA, and IgM. Vγ9Vδ2 T cells also augmented the ability of B cells to stimulate proliferation but not IFN-γ or IL-4 release by alloreactive T cells. In contrast, Vγ9Vδ2 T cells induced expression of CD86 and HLA-DR and the release of IFN-γ, IL-6, and TNF-α by DC and these DC stimulated proliferation and IFN-γ production by conventional T cells. Furthermore, CD86, TNF-α, IFN-γ, and cell contact were found to be important in DC activation by Vγ9Vδ2 T cells but not in the activation of B cells. These data suggest that Vγ9Vδ2 T cells can induce maturation of B cells and DC into APC, but while they prime DC to stimulate T helper 1 (T_H_1) responses, they drive maturation of B cells into APC that can stimulate different T cell responses. Thus, Vγ9Vδ2 T cells can control different arms of the immune system through selective activation of B cells and DC *in vitro*, which may have important applications in immunotherapy and for vaccine adjuvants.

## Introduction

T cells expressing the Vγ9Vδ2 T cell receptor (TCR) comprise the most abundant γδ T cell subset in human blood, where they typically account for 1–5% of T cells in healthy adults (1–4). In many microbial infections, Vγ9Vδ2 T cells dramatically expand, reaching >50% of all T cells at infected sites (5), thus indicating their importance in antimicrobial immunity and their potential for diagnostic and therapeutic use. The Vγ9Vδ2 TCR recognizes a variety of low molecular weight pyrophosphate intermediates of isoprenoid biosynthesis (phosphoantigens), but the most potent phosphoantigen known is (E)-4-hydroxy-3-methyl-but-2-enyl pyrophosphate (HMB-PP), an intermediate of the non-mevalonate pathway that is found in the majority of Gram-negative bacteria, some Gram-positive species and some parasites, such as *Plasmodium falciparum* and *Toxoplasma gondii* (1, 6). Recently, butyrophilin 3A (BTN3A/CD277) was shown to bind to phosphoantigens within cells, resulting in activation of Vγ9Vδ2 T cells (7, 8). HMB-PP can be used to induce *in vitro* expansion and activation of Vγ9Vδ2 T cells (9, 10). Activated Vγ9Vδ2 T cells exhibit a range of effector functions including direct cytotoxicity of infected and tumor cells, the induction of inflammatory and immunoregulatory processes and promotion of the survival, differentiation and activation of monocytes, neutrophils, dendritic cells (DC), αβ T cells, and B cells (1–4).

Recent studies have provided evidence that Vγ9Vδ2 T cells can bridge innate and adaptive immune responses by promoting the differentiation of a number of cell types into antigen-presenting cells (APC). DC are the most potent professional APC. They exist in peripheral tissues as specialized cells for pathogen recognition and uptake by phagocytosis, endocytosis, and pinocytosis, which results in their upregulated expression of antigen-presenting and co-stimulatory molecules, secretion of cytokines, and migration to lymphoid organs where they present antigen to naïve T cells (11, 12). Vγ9Vδ2 T cells, alone and in synergy with pathogen products, can induce differentiation of DC into immunogenic APC that express co-stimulatory markers, produce cytokines and stimulate T cells (10, 13–17). Furthermore, HMB-PP-stimulated Vγ9Vδ2 T cells are also capable of promoting survival and differentiation of monocytes into inflammatory DC (18, 19). Vγ9Vδ2 T cells are also capable of inducing recruitment, activation, and survival of neutrophils (20, 21) and a recent study has shown that neutrophils exposed to Vγ9Vδ2 T cells acquire the ability to present microbial antigens to CD4^+^ T cells and to cross-present endogenous antigens to CD8^+^ T cells (22).

B cells are also capable of presenting antigens to T cells (23) and secreting cytokines that activate and regulate adaptive immune responses (24). A number of studies have demonstrated that Vγ9Vδ2 T cells can induce differentiation of B cells into antibody-producing plasma cells (25–28). They can be found in germinal centers, can acquire features of follicular helper T cells and can induce the production and affinity maturation of class-switched antibodies. However, it is not known if Vγ9Vδ2 T cells contribute to antigen-presentation and cytokine secretion by B cells. The aim of the present study was to investigate the ability of Vγ9Vδ2 T cells to induce differentiation, cytokine secretion, antibody production, and T cell allostimulation by B cells and how this compares to the adjuvant effect of Vγ9Vδ2 T cells for DC. We also examined the requirements for cell contact, co-stimulatory molecule, and cytokine receptor engagement between Vγ9Vδ2 T cells and B cells or DC for their reciprocal stimulatory activities. Our results show that Vγ9Vδ2 T cells induce maturation of both DC and B cells into APC that express co-stimulatory molecules and produce cytokines, and that these mature DC and B cells are capable of inducing alloreactive T cell proliferation. In addition, Vγ9Vδ2 T cell-stimulated B cells secrete antibodies. However, we show that Vγ9Vδ2 T cell-matured DC and B cells have different cytokine profiles and distinct stimulatory capacities for T cells and are mediated by different molecular interactions. Thus, Vγ9Vδ2 T cells can control different effector arms of the immune system through interactions with DC and B cells *in vitro*.

## Materials and Methods

### Donors

Peripheral blood mononuclear cells were prepared from healthy human buffy coat packs obtained from the Irish Blood Transfusion Service (IBTS, St. James’s Hospital, Dublin, Ireland) by standard density gradient centrifugation over Lymphoprep™(Nycomed Pharma, Oslo, Norway). The IBTS provides *pro bono* blood components to Irish third level educational facilities or health care facilities for the purposes of research and education. This blood is from voluntary, anonymous, non-remunerated donors donated primarily for therapeutic application to patients.

### *In vitro* Vδ2 T cell expansion

γδ T cells were enriched from peripheral blood mononuclear cells (PBMC) by positively selecting γδ TCR^+^ cells using a magnetic Microbead cell sorting kit (Miltenyi Biotec, Bergisch-Gladbach, Germany). Vγ9Vδ2 T cells were expanded in 24-well plates by stimulating with 10 nM HMB-PP (kindly provided by Dr. Hassan Jomaa and Dr. Armin Reichenberg) and culturing them in complete RPMI (cRPMI) medium (RPMI 1640 with Glutamax containing 10% heat inactivated fetal calf serum, 50 U/ml penicillin, 50 mg/ml streptomycin, 2 μg/ml fungizone, and 25mM HEPES buffer, Gibco-BRL, Paisley, UK) supplemented with 50 IU/ml IL-2 (Peprotech, New Jersey, USA or Miltenyi Biotec). The medium was changed every 3–4 days by replacing with fresh IL-2-supplemented cRPMI. The cells were harvested on days 14–28 and used for co-culture with DC or B cells. We previously found that virtually all Vδ2^+^ T cells express the Vγ9 chain. Therefore, Vγ9Vδ2 T cells were subsequently identified by a Vδ2 monoclonal Ab (mAb) and are referred to as Vδ2 T cells hereafter (10). Cell purities were determined by staining with mAb against CD3 and Vδ2 and analyzing by flow cytometry.

### B cell isolation

B cells were obtained from human PBMC by positive selection magnetic bead cell sorting (Miltenyi Biotec) of CD19^+^ lymphocytes or by negative selection magnetic bead cell sorting of CD19^−^ lymphocytes (Stemcell Technologies, Canada). The B cells were suspended in cRPMI and used fresh for co-culture with Vδ2 T cells. Purity was determined by staining the cells with anti-CD19 and anti-CD20 mAb and analysis by flow cytometry.

### Dendritic cell preparation

Monocyte-derived DC were obtained from human PBMC by positively selecting CD14^+^ cells (Miltenyi Biotec). The monocytes were induced to differentiate into immature DC by culturing them in DC medium (RPMI 1640 supplemented with 10% heat inactivated, filtered low-endotoxin HyClone fetal calf serum, 1% penicillin-streptomycin, 1% fungizone, 1% L-glutamine, 0.1% β-mercaptoethanol, 1% sodium pyruvate, 1% non-essential amino acid mixture, 1% essential amino acid mixture, and 2% HEPES; Gibco-BRL; Logan, UT, USA) containing IL-4 (70 ng/ml) and GM-CSF (50 ng/ml) (Immunotools, Friesoythe, Germany). After 3 days, medium was replaced with fresh DC medium containing IL-4 and GM-CSF. On day 6, immature DC were harvested and used for co-culture with Vδ2 T cells.

### Antibodies and flow cytometry

Fluorochrome-conjugated human mAb specific for CD3, CD11c, CD14, CD19, CD20, CD40, CD80, CD86, HLA-DR, IFN-γ, IL-4, IL-6, IL-10, IL-12p40, IL-13, TNF-α, and Vδ2 were obtained from Biolegend (San Diego, CA, USA), Immunotools or eBioscience (Hatfield, UK). Fixable viability dye eFluor 506 (eBioscience) was used to determine cell viability. Staining was carried out in PBA buffer (phosphate-buffered saline containing 1% bovine serum albumin and 0.02% sodium azide; Gibco-BRL; Sigma-Aldrich, Ireland) and analyzed using CyAn ADP (Beckman Coulter, High Wycombe, UK) or FACS Canto-II (Becton Dickinson, USA) flow cytometers and FlowJo software (Treestar, Ashland, OR, USA) using fluorescence-minus-one controls. Flow cytometry was used to look at cell surface phenotypes, intracellular cytokines, antibody production, co-stimulatory marker expression, and alloreactive T cell proliferation.

### Analysis of co-stimulatory marker expression by DC and B cells

Vδ2 T cells were cultured with either B cells or DC in equal numbers in the presence or absence of HMB-PP (10 nM) for 72 or 24 h in cRPMI, respectively. The cells were stained for expression of CD11c (DC) or CD19 (B cells) and markers of antigen-presentation CD40, CD80, CD86, and HLA-DR. Surface expression of these markers was compared by mean fluorescence intensity (MFI) readings obtained using flow cytometry.

### Analysis of cytokine release from co-cultures

Vδ2 T cells were cultured with either B cells or DC in equal numbers in the presence or absence of HMB-PP for 72 or 24 h, respectively. The supernatants were then harvested and assayed for levels of IFN-γ, IL-4, IL-6, IL-10, IL-12p70, and TNF-α by enzyme-linked immunosorbent assay (ELISA) using R&D Systems DuoSet kits (Abingdon, UK).

### Analysis of intracellular cytokine production

Vδ2 T cells were cultured with either B cells or DC at 1:1 ratios in the presence or absence of HMB-PP for 24 h and then treated with monensin (10 μl/ml, Biolegend) overnight. The cells were then stained for cell surface expression of CD3 and Vδ2 (Vδ2 T cells), CD19 (B cells), or CD11c (DC). The cells were then fixed and permeabilized and stained for intracellular expression of IFN-γ, IL-4, IL-6, IL-10, IL-12p40, IL-13, and TNF-α for analysis by flow cytometry.

### Measurement of antibody production by B cells

Vδ2 T cells were cultured with B cells at 1:1 ratios in the presence or absence of HMB-PP (10 nM) for 7 days. The supernatants were harvested and analyzed using immunoglobulin cytometric bead array kits (Becton Dickinson) for IgA, IgM, IgE, and total IgG levels.

### Blocking experiments

Vδ2 T cells were cultured with either DC or B cells in equal numbers in the presence or absence of HMB-PP and low-endotoxin, azide-free functional grade blocking antibodies against CD86, CD40L, IFN-γ and IFN-γR, IL-4 and IL-4R, or TNF-α or isotype control mAbs for 24 h. Similar cultures were set up in transwell plates to prevent cell contact. The effects of blocking on DC and B cell phenotypes, cytokine expression and release and antibody production were determined as described above.

### Alloreactive T cell proliferation

Vδ2 T cells were cultured with either B cells or DC in equal numbers in the presence or absence of HMB-PP for 24 h. γδ^−^PBMC were enriched for CD3^+^ cells using a magnetic bead cell sorting kit (Miltenyi Biotec) and stained using a CellTrace™ kit (Invitrogen, CA, USA). The CellTrace-labeled resting alloreactive αβ T cells were added to the overnight culture at ratios of 10:1 or 1:1 and cultured for 6 days before analysis of CellTrace dye dilution of CD3^+^ T cells by flow cytometry. Phytohemagglutinin-P (Sigma-Aldrich)-stimulated αβ T cells cultured with IL-2 and irradiated PBMCs were used as positive controls. Similar co-cultures, except using unlabeled alloreactive αβ T cells, were incubated for 3 days to look for expression of intracellular cytokines by alloreactive T cells. The supernatants harvested on day 3 were assayed for IL-2, IL-4, IL-10, and IFN-γ secretion by ELISA.

### Statistical analysis

GraphPad Prism 5.0 (San Diego, CA, USA) was used to carry out paired and unpaired *t*-tests to compare the means between groups. *P* values of <0.05 were considered statistically significant.

## Results

### Vδ2 T cells induce APC marker expression by DC and B cells

**Figure 1:**
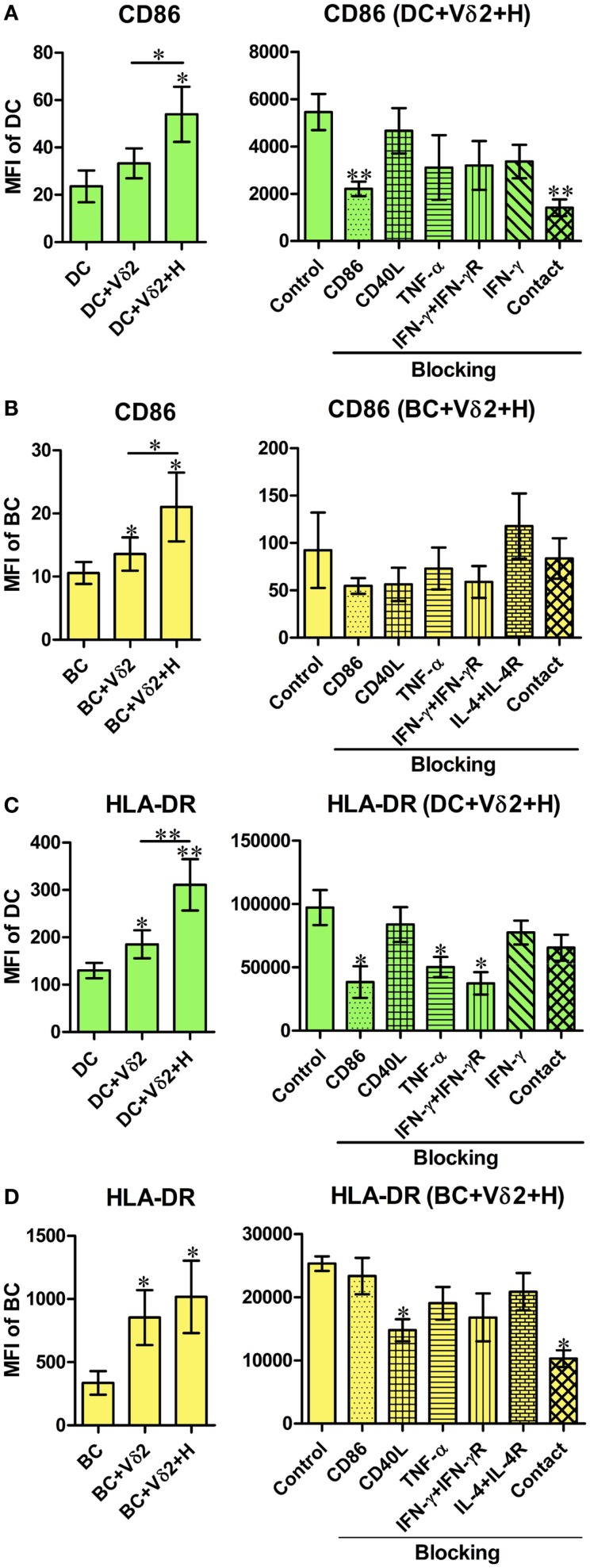
**Vδ2 T cells induce the expression of APC markers by dendritic cells (DC) and B cells (BC)**. Monocyte-derived DC or enriched peripheral blood B cells were co-cultured for 24 or 72 h with HMB-PP-expanded human Vδ2 T cells in the absence or presence of HMB-PP (denoted H). Cells were then stained using mAb specific for CD11c or CD19 and CD86 and HLA-DR and analyzed by flow cytometry. Left panels show average (±SEM) mean fluorescence intensities (MFI) of staining for CD86 expression by **(A)** DC (*n* = 11) and **(B)** B cells (*n* = 12) and HLA-DR expression by **(C)** DC (*n* = 9) and **(D)** B cells (*n* = 7). Right panels show average (±SEM) MFI of staining for CD86 or HLA-DR by DC or B cells after co-culturing them with Vδ2 T cells in the presence of HMB-PP in the absence (control) or presence of blocking mAbs specific for CD86, CD40L, TNF-α, IFN-γ + IFN-γR, IL-4 + IL-4R or with the DC or B cells separated from Vδ2 T cells using transwell inserts (*n* = 5 for DC treatments and *n* = 3 for BC treatments). *p < 0.05, **p < 0.01 using a paired *t*-test, compared to DC or BC alone (left panels) or compared to BC control (right panels) and unpaired *t*-test compared to DC control (right panels) except where indicated by horizontal lines.

We initially investigated if Vδ2 T cells can induce differentiation of B cells into cells with phenotypes of APC. Therefore, we examined the expression of CD40, CD86, and HLA-DR by B cells or DC after co-culture with non-stimulated or HMB-PP-activated Vδ2 T cells. Vδ2 T cells induced an increase in CD86 (Figure 1A) and HLA-DR (Figure 1C), but not CD40 (Figure S1A in Supplementary Material) expression by DC after 24 h and CD86 (Figure 1B), HLA-DR (Figure 1D) and CD40 (Figure S1B in Supplementary Material) expression by B cells after 72 h. CD86 expression was also upregulated on B cells after 24 h. To investigate which molecules are involved in DC and B cell activation by Vδ2 T cells or whether it is cell contact dependent, the same co-cultures were set up in the presence of HMB-PP-activated Vδ2 T cells and one of several blocking antibodies or transwell inserts which prevent cell contact between the different cell types in the co-cultures. The results show that cell contact is important for CD86 expression by DC (Figure 1A), while CD86, TNF-α, and IFN-γ are important for HLA-DR expression by DC (Figure 1C). In contrast, CD40L and cell contact are important for HLA-DR expression (Figure 1D) but not CD40 expression (Figure S1B in Supplementary Material) by Vδ2-stimulated B cells.

### Vδ2 T cells induce distinct cytokine expression by DC and B cells

**Figure 2:**
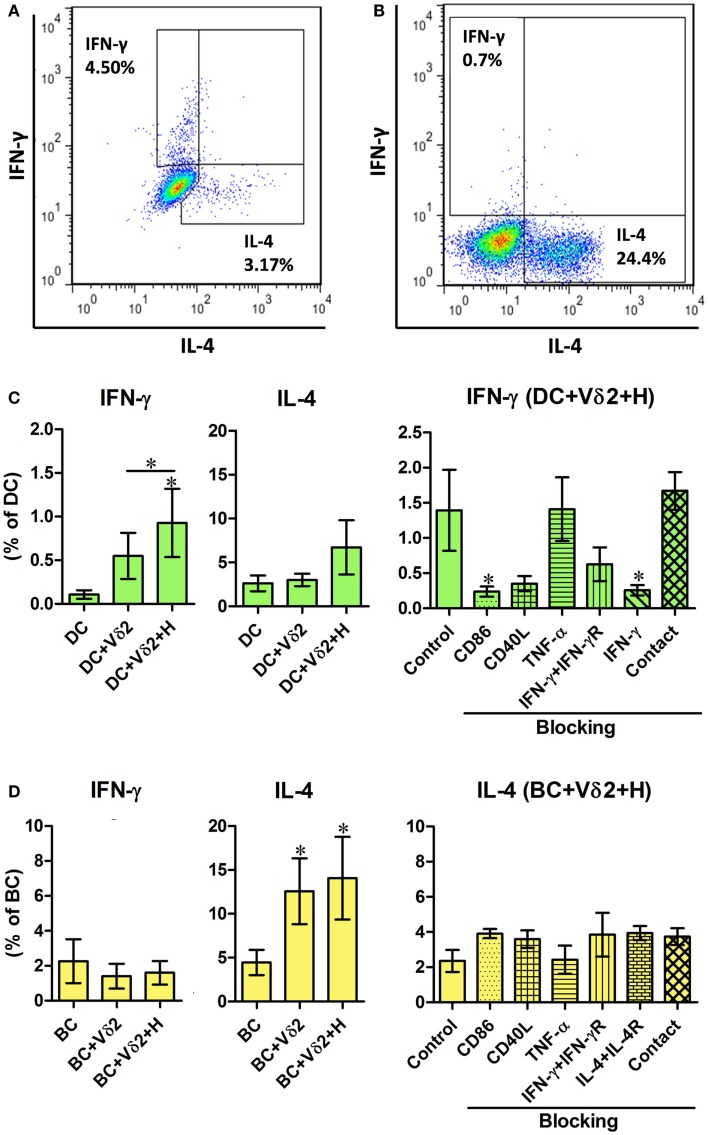
**Vδ2 T cells induce distinct cytokine profiles by DC and B cells**. DC or B cells were co-cultured with HMB-PP-expanded human Vδ2 T cells in the absence or presence of HMB-PP (denoted H) for 24 h. The cultures were then treated with monensin for a further 16 h and stained for cell surface expression of CD11c or CD19 and CD3 and Vδ2 and intracellular expression of IFN-γ or IL-4 and analyzed by flow cytometry. **(A,B)** Representative flow cytometric dot plots showing IFN-γ and IL-4 expression by gated CD11c^+^ cells (DC) and CD19^+^ cells (BC), respectively. **(C,D)** Left and center panels show mean (±SEM) percentages of **(C)** DC (*n* = 10) and **(D)** BC (*n* = 10) that express IFN-γ and IL-4, respectively. Right panels show mean (±SEM) percentages of **(C)** DC and **(D)** BC expressing IFN-γ and IL-4, respectively, after co-culturing them with Vδ2 T cells in the presence of HMB-PP in the absence (control) or presence of blocking mAbs specific for CD86, CD40L, TNF-α, IFN-γ + IFN-γR, IL-4 + IL-4R or with the DC (*n* = 5), or BC (*n* = 3) separated from Vδ2 T cells using transwell inserts. *p < 0.05 using a paired *t*-test, compared to DC or BC alone (left panels) or compared to BC control (right panels) and unpaired *t*-test compared to DC control (right panels) except where indicated by horizontal lines.

To further characterize the influence of Vδ2 T cells on DC and B cell activation, we examined the same co-cultures for intracellular cytokine expression. The co-cultures, as described above, were treated with monensin for 16 h and the DC or B cells were analyzed for intracellular IFN-γ, IL-4 (Figures 2A,B), and TNF-α (Figure S2 in Supplementary Material) expression by flow cytometry. Vδ2 T cells induced IFN-γ expression by DC (Figure 2C) but not B cells and IL-4 expression by B cells (Figure 2D) but not DC. In contrast, Vδ2 T cells induced TNF-α expression by both DC and B cells (Figure S2 in Supplementary Material). The blocking studies revealed that CD86 and IFN-γ are important for IFN-γ expression by DC (Figure 2C), but not for cytokine production by B cells (Figure 2D).

### Vδ2 T cells induce pro- and anti-inflammatory cytokine secretion from DC and B cell co-cultures

**Figure 3:**
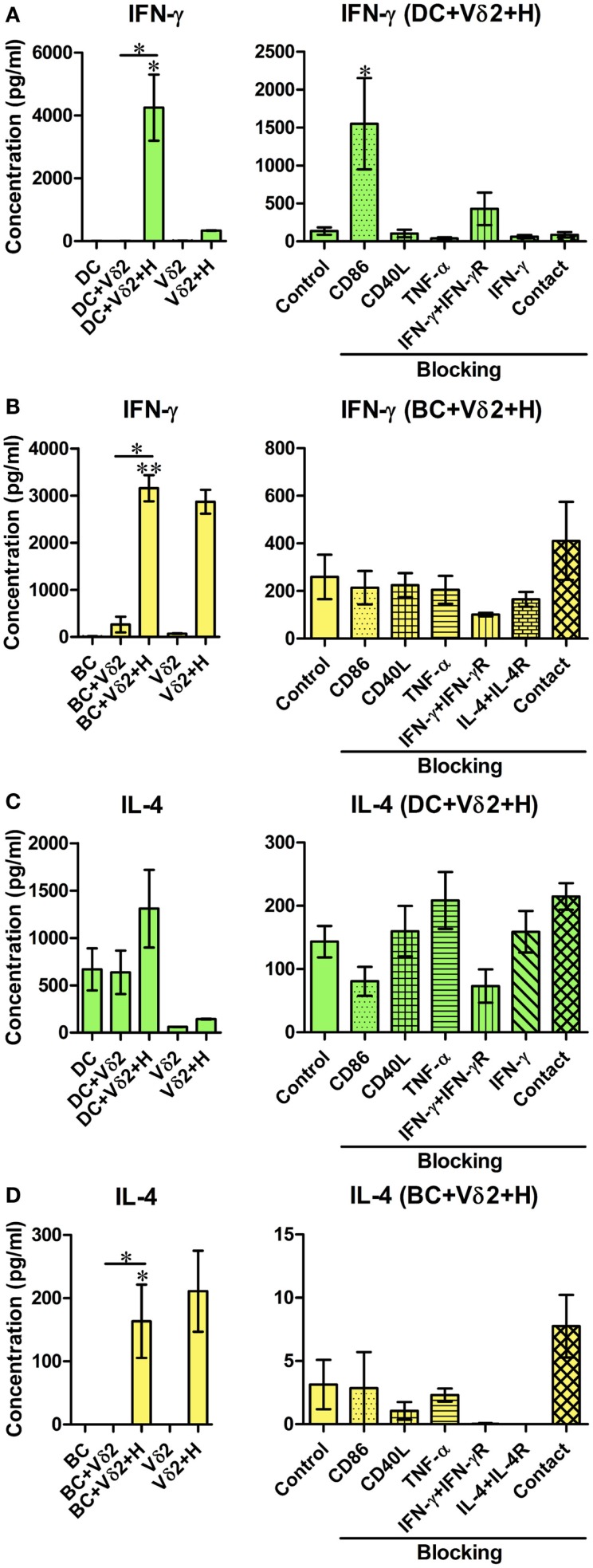
**Co-cultures of Vδ2 T cells and DC or B cells have distinct cytokine secretion profiles**. DC or B cells were co-cultured with HMB-PP-expanded human Vδ2 T cells in the absence or presence of HMB-PP (denoted H). After 24 h (for DC) or 72 h (for B cells), supernatants were harvested and analyzed for IFN-γ and IL-4 by ELISA. Left panels show mean (±SEM) concentration of IFN-γ in **(A)** DC (*n* = 3) and **(B)** BC (*n* = 3) co-cultures and IL-4 in **(C)** DC (*n* = 3) and **(D)** BC (*n* = 6) co-cultures. Right panels show average (±SEM) concentration of IFN-γ and IL-4 from the DC and B cell co-cultures in the presence of HMB-PP in the absence (control) or presence of blocking mAb specific for CD86, CD40L, TNF-α, IFN-γ + IFN-γR, IL-4 + IL-4R or with the DC (*n* = 5), or B cells (*n* = 3) separated from Vδ2 T cells using transwell inserts. *p < 0.05, **p < 0.01 Using a paired *t*-test, compared to DC or BC alone (left panels) or compared to BC control (right panels) and unpaired *t*-test compared to DC control (right panels) except where indicated by horizontal lines.

While the flow cytometric cytokine assay revealed the percentage of cells expressing cytokines, we wanted to quantify the levels of cytokine production from the co-cultures. After 24 h co-culture of Vδ2 T cells and DC or B cells, supernatants were analyzed for levels of IFN-γ, TNF-α, IL-4, IL-6, IL-10, and IL-12 by ELISA. Since the cellular source of the cytokines produced cannot be identified, we also examined cytokine production by Vδ2 T cells alone. We found that Vδ2-DC co-cultures produced IFN-γ (Figure 3A), TNF-α (not shown), and IL-6 (Figure S3A in Supplementary Material) but not IL-4 (Figure 3C), IL-10, or IL-12 (Figure S3A in Supplementary Material) after 24 h. In contrast, Vδ2-B cell co-cultures produced TNF-α and IL-6 but did not augment IFN-γ (Figure 3B), IL-4 (Figure 3D), IL-10, or IL-12 (Figure S3B in Supplementary Material) production compared with Vδ2 T cells cultured alone. IFN-γ production by HMB-PP-activated Vδ2 T cells was also observed by flow cytometry (data not shown). None of the molecules tested in the blocking studies, nor cell contact were found to be important for cytokine secretion by these co-cultures. However, surprisingly, blocking of CD86 resulted in augmented IFN-γ secretion after co-culture with Vδ2 T cells.

### Vδ2 T cells induce antibody production by B cells

**Figure 4:**
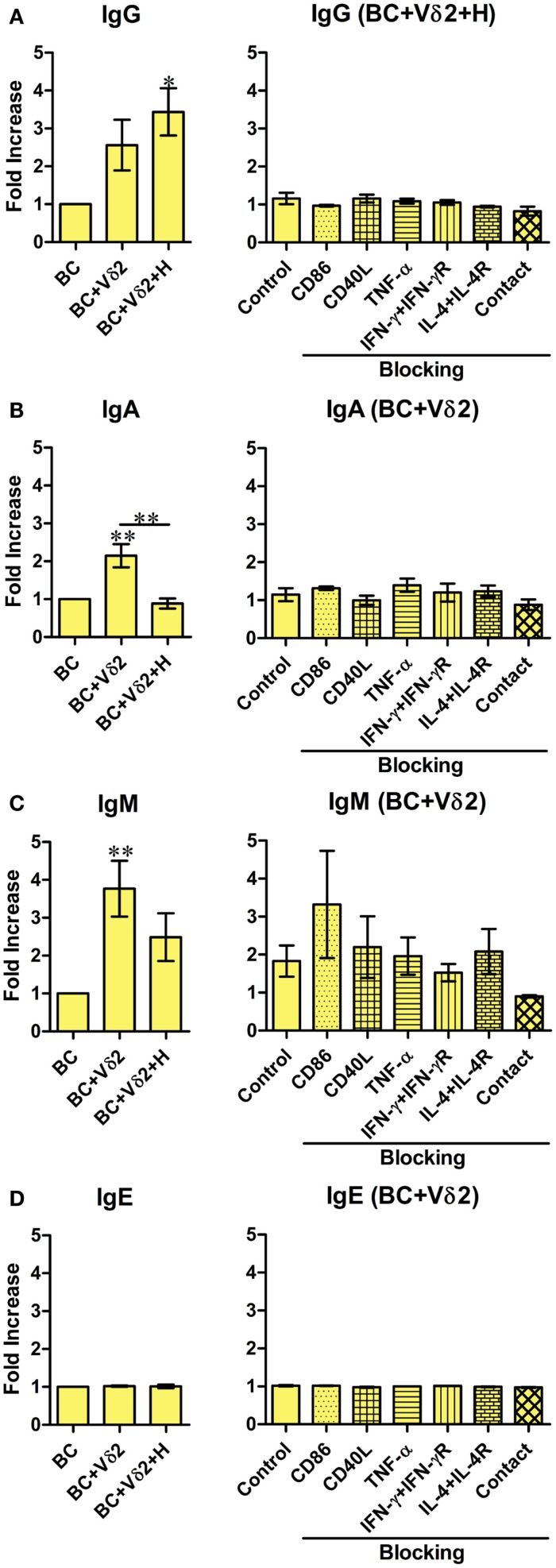
**Vδ2 T cells induce antibody production by B cells**. B cells were co-cultured with HMB-PP-expanded human Vδ2 T cells in the absence or presence of HMB-PP (denoted H). After 7 days the supernatants were harvested and analyzed for IgA, IgM, IgE, and total IgG levels by cytometric bead array and flow cytometry. Left panels show average mean (±SEM) MFI of staining for **(A)** IgG (*n* = 5), **(B)** IgA (*n* = 8), **(C)** IgM (*n* = 7), and **(D)** IgE (*n* = 2). Right panels show average (±SEM) MFI intensities of IgG, IgA, IgM, and IgE of B cells after co-culturing them with Vδ2 T cells in the presence of HMB-PP in the absence (control) or presence of blocking mAbs specific for CD86, CD40L, TNF-α, IFN-γ + IFN-γR, IL-4 + IL-4R, or with the B cells separated from Vδ2 T cells using transwell inserts (*n* = 3). *p < 0.05, **p < 0.01 using a paired *t*-test, compared to BC alone (left panels) or compared to B cell control (right panels) except where indicated by horizontal lines.

Previous studies have shown that a subset of Vδ2 T cells can provide help for antibody production by B cells and that it was mediated by CD40L, ICOS, and IL-10 (28). To investigate whether Vδ2 T cells can induce immunoglobulin production by fresh peripheral B cells *in vitro*, Vδ2 T cells were cultured with B cells for 7 days, and the supernatants were analyzed for total IgG, IgA, IgM, and IgE by a flow cytometric bead array. Vδ2 T cells induced IgG (Figure 4A), IgA (Figure 4B), IgM (Figure 4C) but not IgE (Figure 4D) production by B cells, while HMB-PP-activated Vδ2 T cells prevented IgA (Figure 4B) and IgM (Figure 4C) production. The blocking studies revealed that the cytokines and co-stimulatory markers examined and cell contact, do not play a part in antibody production by B cells.

### Vδ2-matured DC and B cells stimulate proliferation of resting allogeneic T cells

**Figure 5:**
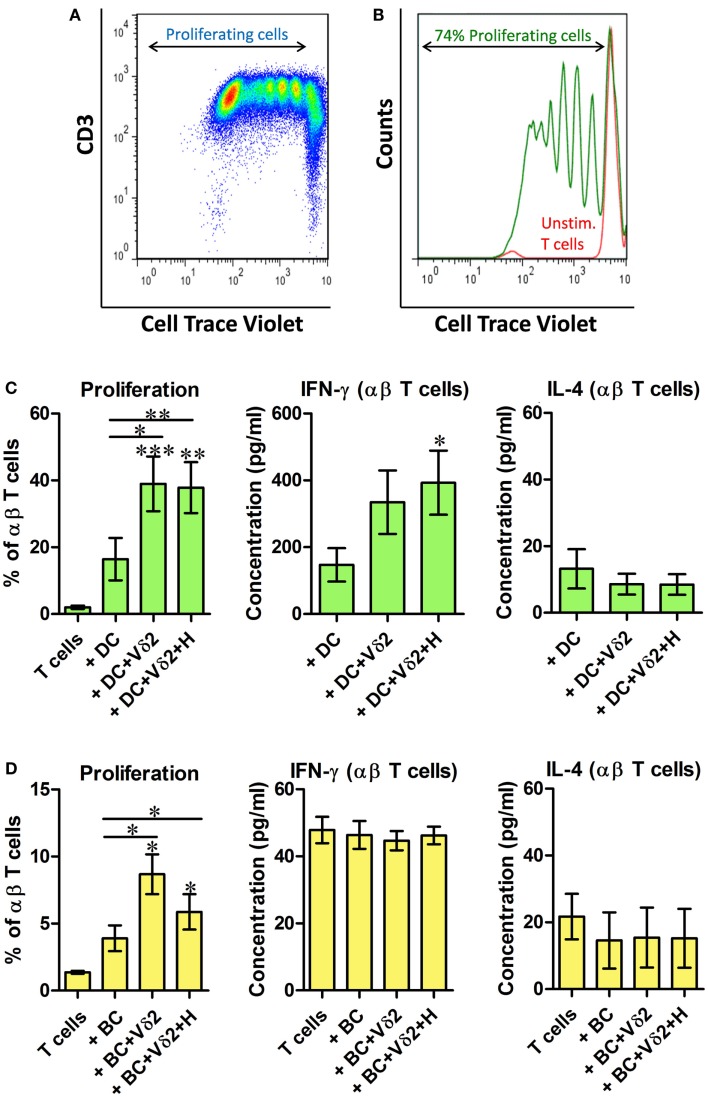
**Vδ2-matured DC and B cells stimulate proliferation of resting allogeneic T cells**. DC or B cells were co-cultured with HMB-PP-expanded human Vδ2 T cells in the absence or presence of HMB-PP (denoted H). After 24 h CellTrace-labeled allogeneic resting αβ T cells were added at a ratio of 10:1 and cultured for 6 days. **(A)** Representative dot plot showing proliferating T cells. **(B)** Histogram showing proliferating T cells (green peaks) versus unstimulated T cells (red peak) by flow cytometric analysis of cell trace dilution. **(C)** Average (±SEM) percentage of proliferating T cells when cultured with Vδ2-matured DC (*n* = 10; left) and levels of IFN-γ and IL-4 secreted by cultures of Vδ2 T cell matured DC with αβ T cells (*n* = 6–10). **(D)** Average (±SEM) percentage of proliferating T cells when cultured with Vδ2-matured B cells (*n* = 4) and levels of IFN-γ and IL-4 secreted by cultures of Vδ2 T cell matured DC with αβ T cells (*n* = 4). *p < 0.05, **p < 0.01, ***p < 0.001, paired *t*-test versus T cells except where indicated by horizontal lines.

We investigated whether Vδ2 T cell-matured DC and B cells can induce activation and proliferation of resting αβ T cells. Vδ2 T cell-matured DC or B cells were cultured with 10 times as many CellTrace-labeled resting allogeneic αβ T cells for 6 days and dye dilution due to cell proliferation was examined by flow cytometry (Figures 5A,B). The co-cultures showed that both DC (Figure 5C) and B cells (Figure 5D) induced activation and proliferation of resting T cells after co-culture with Vδ2 T cells. Similar 3 day co-cultures were set up for analysis of cytokine secretion. ELISA showed that Vδ2 T cell-matured DC induced IFN-γ but not IL-4 production by T cells, whereas Vδ2 T cell-matured B cells did not stimulate cytokine production by T cells (Figures 5C,D; Figure S5 in Supplementary Material).

### Allogeneic and autologous Vδ2 T cells equally activate DC and B cells

The experiments described above indicate that Vδ2 T cells can differentially induce MHC and co-stimulatory molecule expression, cytokine production, and T cell allostimulation by allogeneic DC and B cells. We also investigated if the same outcomes could be observed when Vδ2 T cells were cultured with autologous DC or B cells. Figure S4 in Supplementary Material shows that Vδ2 T cells could equally induce CD86 expression (Figure S4A in Supplementary Material) and IL-12 secretion (Figure S4B in Supplementary Material) by autologous and allogeneic DC, and CD86 expression (Figure S4C in Supplementary Material) and IL-4 secretion (Figure S4D in Supplementary Material) by autologous and allogeneic B cells. Thus it appears that allogeneic Vδ2 T cells can be substituted for autologous Vδ2 cells as adjuvants for DC or B cells.

## Discussion

Vγ9Vδ2 T cells exhibit a myriad of effector functions in innate and adaptive immunity. They can kill infected, tumor, and stressed target cells, promote inflammation and wound healing, promote the survival, differentiation and activation of monocytes, neutrophils, and DC, provide B cell help for antibody production and prime CD4^+^ and CD8^+^ T cells (1–4). Vγ9Vδ2 T cells can also link innate and adaptive immune responses by promoting differentiation of different types of cells into APC that are capable of initiating antigen-specific T cell responses and long-term immunological memory (10, 13–19, 22, 29). These findings implicate Vγ9Vδ2 T cells as candidate targets for development of novel therapies and vaccines.

The findings in the present study confirm previous reports that Vγ9Vδ2 T cells can induce maturation, MHC and co-stimulatory receptor expression, and T_H_1 cytokine production by DC (10, 13–17) and further show that these matured DC can stimulate proliferation and T_H_1 cytokine production by alloreactive αβ T cells. We found that Vδ2-DC co-cultures secreted IFN-γ, TNF-α, and IL-6 but not IL-4 and IL-10 after 24 h. While Vδ2 T cells were not potent inducers of IL-12 production by DC, they exhibited a strong synergistic effect with TLR ligands, such as LPS in inducing IL-12 release. Importantly, DC matured with Vδ2 T cells could stimulate proliferation and IFN-γ production by resting alloreactive T cells *in vitro*, suggesting that these APC also prime antigen-specific T_H_1 responses. Although we did not test if Vδ2 T cell-matured DC could present specific antigen to T cells, their ability to stimulate alloreactive T cells to a greater degree than DC that had not been cultured with Vδ2 T cells, suggests that Vδ2 T cells are promoting differentiation of DC into APC.

Previous studies have demonstrated that Vγ9Vδ2 T cells can induce maturation of B cells into antibody-secreting plasma cells (25–28), suggesting that they can promote humoral immune responses *in vivo*. We showed that HMB-PP-stimulated Vδ2 T cells can stimulate the production of IgG, IgM, and IgA but not IgE by B cells *in vitro* and that HMB-PP prevents IgM and IgA production. We also examined the phenotypic changes to B cells that occur in response to co-culturing them with Vδ2 T cells and found that, like for DC, B cells upregulated HLA-DR, CD40, and CD86, suggesting that Vδ2 T cells can drive maturation of B cells into APC. However, analysis of cytokine production revealed that Vδ2-B cell co-cultures could produce TNF-α, IL-6, and IL-4 but not IFN-γ or IL-12. Thus Vδ2-matured DC and B cells have distinct cytokine profiles, with B cells lacking the T_H_1-promoting cytokine bias seen for DC. Analysis of the capacity of Vδ2 T cell-matured B cells to stimulate alloreactive T cells indicated that they could induce proliferation but not IFN-γ, IL-2, IL-4, or IL-10 production. These findings suggest that Vδ2 T cells can drive the differentiation of DC into T_H_1-promoting APC and B cells into APC that can stimulate different T cell responses.

Several studies have demonstrated a flexibility of DC maturation and their ability to differentiate into APC that selectively promote T_H_1, T_H_2, or tolerogenic T cell responses (30–33). The factors that determine the fate of DC differentiation include the nature of antigen and the presence of TLR ligands and cytokines and it appears that Vγ9Vδ2 T cells contribute by driving T_H_1-promoting APC generation. Tolerogenic APC are characterized by the expression of MHC class II and co-stimulatory molecules in the absence of pro-inflammatory cytokine production and they can present antigen to T cells resulting in the induction of anergy or the expansion of regulatory T cells (30–33). Our data suggest that Vδ2 T cell-matured B cells may function as tolerogenic APC, since they display phenotypes of APC but they do not produce pro-inflammatory cytokines and they stimulate proliferation but not cytokine production by alloreactive T cells. Furthermore, the ability of Vδ2-matured B cells to produce the anti-inflammatory cytokine IL-4 further supports a tolerogenic phenotype and we speculate that the IL-4 may function in promoting antibody responses. This is supported by the study by Caccamo (26), which showed that a subset of Vδ2 T cells that produce IL-4 and IL-10 provide help to B cells for antibody production. B cells have previously been shown to present antigen, resulting in tolerogenic T cell responses (34, 35), but future work is required to determine if the T cells stimulated by Vδ2-matured B cells have tolerogenic or immunosuppressive activities.

Since the mechanisms underlying DC and B cell activation by Vδ2 T cells are poorly understood, we aimed to identify the molecules required to mediate these functional changes. We found that while co-stimulatory molecules, pro-inflammatory cytokines and physical contact with Vδ2 T cells were important for DC maturation, co-stimulatory markers, and contact played only a minor role in B cell maturation and were not important for antibody production. Blocking CD40L and separating the B cells from Vδ2 T cells resulted in less upregulation of HLA-DR by B cells, but did not significantly affect the other readouts. Our results are in contrast to the study by Caccamo (26), which showed that IL-10, IL-4, CD40L, and ICOS abrogated antibody production. However, they did not investigate the role of these factors on co-stimulatory marker expression and cytokine production by B cells. Thus, the mechanisms responsible for B cell activation need to be further elucidated.

**Figure 6:**
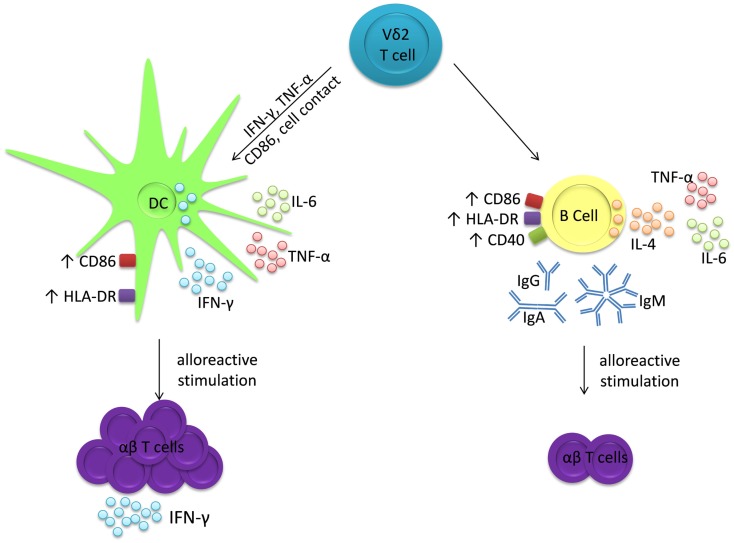
**Vδ2 T cells have distinct adjuvant effects for DC and B cells**. Co-cultures of Vδ2 T cells with either DC or B cells produce IL-6 and TNF-α, but not IL-12 nor IL-10. However, Vδ2 T cells induce IFN-γ production and co-stimulatory marker expression by DC after 24 h and these matured DC induce proliferation of alloreactive T cells, which then secrete IFN-γ. In contrast, Vδ2 T cells induce IL-4 production and co-stimulatory molecule expression by B cells after 72 h and immunoglobulin secretion after 7 days. These matured B cells induce proliferation but not IFN-γ, IL-2, IL-4 nor IL-10 production by alloreactive T cells.

The adjuvant effects of Vδ2 T cells display similarities to those of other innate T cells. Invariant natural killer T (iNKT) cells, so called because of their conserved TCR α-chains that recognize glycolipid antigens presented by CD1d, can also induce maturation of DC into APC (36–38) and B cells into antibody-secreting plasma cells (39–42). Similar to Vδ2 T cells, iNKT cells induce MHC and co-stimulatory molecule expression by both DC and B cells, but they predominantly induce IL-12 production by DC (36, 37, 43) and IL-4 and IL-10 production by B cells (44). Furthermore, DC cultured with iNKT cells acquire phenotypes and functions of immunogenic APCs, whereas B cells cultured with iNKT cells differentiate into antibody-producing plasma cells but they are inhibited in their ability to stimulate alloreactive T cell proliferation (44). Other subsets of human γδ T cells also possess adjuvant activities. T cells expressing the Vδ1 and Vδ3 TCRs can promote maturation of DC into APCs capable of driving T cell proliferation (45–47) and one study has shown that a population of Vδ1^+^ T cells specific for pollen-derived antigens can drive IgE production by B cells *in vitro* (48). Therefore, Vδ2 T cells belong to a family of innate T cells that can differentially promote or regulate T cell and antibody responses through selective interactions with DC and B cells. Whereas Vδ2 T cells promote immunogenic T_H_1 responses by inducing maturation of DC into APCs, they appear to promote T cell tolerance via their adjuvant activities on B cells, while at the same time promoting antibody production (Figure 6). While Vδ2 T cells are already under investigation as adjuvants for immunotherapies in clinical trials for cancer (49–51), their distinct effects on DC and B cells must be considered in order to prevent unwanted immunosuppression or autoimmunity.

## Conflict of Interest Statement

The authors declare that the research was conducted in the absence of any commercial or financial relationships that could be construed as a potential conflict of interest.

## Supplementary Material

The Supplementary Material for this article can be found online at http://www.frontiersin.org/Journal/10.3389/fimmu.2014.00650/abstract

## References

[1] Morita CT Jin C Sarikonda G Wang H. Nonpeptide antigens, presentation, mechanisms and immunological memory of human Vγ2Vδ2 T cells: discriminating friend from foe through the recognition of prenyl pyrophosphate antigens. Immunol Rev (2007) 215:59–76. 10.1111/j.1600-065X.2006.00479.x 17291279

[2] Kabelitz D He W. The malfunctionality of human Vγ9Vδ2 γδ T cells: clonal plasticity or distinct subsets? Scand J Immunol (2012) 76:213–22. 10.1111/j.1365-3083.2012.02727.x 22670577

[3] Vantourout P Hayday AC. Six-of-the-best: unique contributions of γδ T cells to immunology. Nat Rev Immunol (2013) 13:88–100. 10.1038/nri3384 23348415 PMC3951794

[4] Chien YH Meyer C Bonneville M. γδ T cells: first line of defense and beyond. Annu Rev Immunol (2014) 32:121–55. 10.1146/annurev-immunol-032713-120216 24387714

[5] Hara T Mizuno Y Takaki K Takada H Akeda H Aoki T Predominant activation and expansion of Vγ9-bearing γδ T cells in vivo as well as in vitro in Salmonella infection. J Clin Invest (1992) 90:204–10. 10.1172/JCI115837 1386086 PMC443082

[6] Eberl M Hintz M Reichenberg A Kollas AK Wiesner J Jomaa H Microbial isoprenoid biosynthesis and human γδ T cell activation. FEBS Lett (2003) 554:4–10 10.1016/S0014-5793(03)00483-6 12782281

[7] Harly C Guillaume Y Nedellec S Peigné CM Mönkkönen J Li J Key implication of CD277/butyrophilin-3 (BTN3A) in cellular stress sensing by a major human γδ T-cell subset. Blood (2012) 120:2269–79. 10.1182/blood-2012-05-430470 22767497 PMC3679641

[8] Sandstrom A Peigné CM Léger A Crooks JE Konczak F Gesnel MC The intracellular B30.2 domain of butyrophilin 3A1 binds phosphoantigens to mediate activation of human Vγ9Vδ2 T cells. Immunity (2014) 40:490–500. 10.1016/j.immuni.2014.03.003 24703779 PMC4028361

[9] Hintz M Reichenberg A Altincicek B Bahr U Gschwind RM Kollas AK Identification of (E)-4-hydroxy-3-methyl-but-2-enyl pyrophosphate as a major activator for human γδ T cells in Escherichia coli. FEBS Lett (2001) 509:317–22. 10.1016/S0014-5793(01)03191-X 11741609

[10] Dunne MR Madrigal-Estebas L Tobin LM Doherty DG. E(H)-4-hydroxy-3-methyl-but-2 enyl pyrophosphate-stimulated Vγ9Vδ2 T cells possess T helper type 1-promoting adjuvant activity for human monocyte-derived dendritic cells. Cancer Immunol Immunother (2010) 59:1109–20. 10.1007/s00262-010-0839-8 20306041 PMC11030662

[11] Banchereau J Briere F Caux C Davoust J Lebcque S Liu YJ Immunobiology of dendritic cells. Annu Rev Immunol (2000) 18:767–811 10.1146/annurev.immunol.18.1.767 10837075

[12] Mellman I Steinman RM Dendritic cells: specialized and regulated antigen processing machines. Cell (2001) 106:255–8 10.1016/S0092-8674(01)00449-4 11509172

[13] Ismaili J Olislagers V Poupot R Fournié JJ Goldman M. Human γδ T cells induce dendritic cell maturation. Clin Immunol (2002) 103:296–302. 10.1006/clim.2002.5218 12173304

[14] Conti L Casetti R Cardone M Varano B Martino A Belardelli F Reciprocal activating interaction between dendritic cells and pamidronate-stimulated T cells: role of CD86 and inflammatory cytokines. J Immunol (2005) 174:252–60. 10.4049/jimmunol.174.1.252 15611247

[15] Martino A Casetti R D’Alessandri A Sacchi A Poccia F. Complementary function of gamma delta T-lymphocytes and dendritic cells in the response to isopentenyl-pyrophosphate and lipopolysaccharide antigens. J Clin Immunol (2005) 25:230–7. 10.1007/s10875-005-4080-8 15981088

[16] Shrestha N Ida JA Lubinski AS Pallin M Kaplan G Haslett PA. Regulation of acquired immunity by γδ T-cell/dendritic-cell interactions. Ann N Y Acad Sci (2005) 1062:79–94. 10.1196/annals.1358.011 16461791

[17] Devilder MC Maillet S Bouyge-Moreau I Donnadieu E Bonneville M Scotet E. Potentiation of antigen-stimulated Vγ9Vδ2 T cell cytokine production by immature dendritic cells (DC) and reciprocal effect on DC maturation. J Immunol (2006) 176:1386–93. 10.4049/jimmunol.176.3.1386 16424165

[18] Kalyan S Chow AW. Linking innate and adaptive immunity: human Vγ9Vδ2 T cells enhance CD40 expression and HMGB-1 secretion. Mediators Inflamm (2009) 2009:819408. 10.1155/2009/819408 19841752 PMC2762119

[19] Eberl M Roberts GW Meuter S Williams JD Topley N Moser B. A rapid crosstalk of human γδ T cells and monocytes drives the acute inflammation in bacterial infections. PLoS Pathog (2009) 5:e1000308. 10.1371/journal.ppat.1000308 19229322 PMC2637987

[20] Agrati C Cimini E Sacchi A Bordoni V Gioia C Casetti R Activated Vγ9Vδ2 T cells trigger granulocyte functions via MCP-2 release. J Immunol (2009) 182:522–9. 10.4049/jimmunol.182.1.522 19109184

[21] Davey MS Lin CY Roberts GW Heuston S Brown AC Chess JA Human neutrophil clearance of bacterial pathogens triggers anti-microbial γδ T cell responses in early infection. PLoS Pathog (2011) 7:e1002040. 10.1371/journal.ppat.1002040 21589907 PMC3093373

[22] Davey MS Morgan MP Liuzzi AR Tyler CJ Khan MW Szakmany T Microbe-specific unconventional T cells induce human neutrophil differentiation into antigen cross-presenting cells. J Immunol (2014) 193:3704–16. 10.4049/jimmunol.1401018 25165152 PMC4169984

[23] Rodriguez-Pinto D B cells as antigen presenting cells. Cell Immunol (2005) 238:67–75 10.1016/j.cellimm.2006.02.005 16574086

[24] Harris DP Haynes L Sayles PC Duso DK Eaton SM Lepak NM Reciprocal regulation of polarized cytokine production by effector B cells and T cells. Nat Immunol (2000) 1:475–82. 10.1038/82717 11101868

[25] Brandes M Willimann K Lang AB Nam KH Jin C Brenner MB Flexible migration program regulates γδ T-cell involvement in humoral immunity. Blood (2003) 102:3693–701. 10.1182/blood-2003-04-1016 12881309

[26] Caccamo N Battistini L Bonneville M Poccia F Fournié JJ Meraviglia S CXCR5 identifies a subset of Vγ9Vδ2 T cells which secrete IL-4 and IL-10 and help B cells for antibody production. J Immunol (2006) 177:5290–5. 10.4049/jimmunol.177.8.5290 17015714

[27] Bansal RR Mackay CR Moser B Eberl M. IL-21 enhances the potential of human γδ T cells to provide B-cell help. Eur J Immunol (2011) 42:110–9. 10.1002/eji.201142017 22009762

[28] Caccamo N Todaro M La Manna MP Sireci G Stassi G Dieli F. IL-21 regulates the differentiation of a human γδ T cell subset equipped with B cell helper activity. PLoS One (2012) 7:e41940. 10.1371/journal.pone.0041940 22848667 PMC3405033

[29] Marcu-Malina V Balbir-Gurman A Dardik R Braun-Moscovici Y Segel MJ Bank I. A novel prothrombotic pathway in systemic sclerosis patients: possible role of biosphosphonate-activated γδ T cells. Front Immunol (2014) 5:414. 10.3389/fimmu.2014.00414 25250025 PMC4157565

[30] Tisch R Immunogenic versus tolerogenic dendritic cells: a matter of maturation. Int Rev Immunol (2010) 29:111–8 10.3109/08830181003602515 20367138

[31] Steinman RM Hawiger D Nussenzweig MC. Tolerogenic dendritic cells. Annu Rev Immunol (2003) 21:685–711. 10.1146/annurev.immunol.21.120601.141040 12615891

[32] Reis e Sousa C. Dendritic cells in a mature age. Nat Rev Immunol (2006) 6:476–83. 10.1038/nri1845 16691244

[33] Lutz MB Schuler G. Immature, semi-mature and fully mature dendritic cells: which signals induce tolerance or immunity? Trends Immunol (2002) 23:445–9. 10.1016/S1471-4906(02)02281-0 12200066

[34] Eynon EE Parker DC. Small B cells as antigen-presenting cells in the induction of tolerance to soluble protein antigen. J Exp Med (1992) 175:131–8. 10.1084/jem.175.1.131 1730913 PMC2119092

[35] Fuchs EJ Matzinger P. B cells turn off virgin but not memory T cells. Science (1992) 258:1156–9. 10.1126/science.1439825 1439825

[36] Kitamura H Iwakabe K Yahata T Nishimura S Ohta A Ohmi Y The natural killer T (NKT) cell ligand alpha-galactosylceramide demonstrates its immunopotentiating effect by inducing interleukin (IL)-12 production by dendritic cells and IL-12 receptor expression on NKT cells. J Exp Med (1999) 189:1121–8. 10.1084/jem.189.7.1121 10190903 PMC2193012

[37] Vincent MS Leslie DS Gumperz JE Xiong X Grant EP Brenner MB. CD1-dependent dendritic cell instruction. Nat Immunol (2002) 3:1163–8. 10.1038/ni851 12415264

[38] Fujii S Shimizu K Smith C Bonifaz L Steinman RM. Activation of natural killer T cells by α-galactosylceramide rapidly induces the full maturation of dendritic cells in vivo and thereby acts as an adjuvant for combined CD4 and CD8 T cell immunity to a coadministered protein. J Exp Med (2003) 198:267–79. 10.1084/jem.20030324 12874260 PMC2194082

[39] Galli G Nuti S Tavarini S Galli-Stampino L De Lalla C Casorati G CD1d-restricted help to B cells by human invariant natural killer T lymphocytes. J Exp Med (2003) 197:1051–7. 10.1084/jem.20021616 12695492 PMC2193881

[40] Devera T Shah H Lang G Lang M. Glycolipid-activated NKT cells support the induction of persistent plasma cell responses and antibody titers. Eur J Immunol (2008) 38:1001–11. 10.1002/eji.200738000 18350547 PMC3500907

[41] Barral P Eckl-Dorna J Harwood NE De Santo C Salio M Illarionov P B cell receptor-mediated uptake of CD1d-restricted antigen augments antibody responses by recruiting invariant NKT cell help in vivo. Proc Natl Acad Sci USA (2008) 105:8345–50. 10.1073/pnas.0802968105 18550831 PMC2448839

[42] Leadbetter E Brigl M Illarionov P Cohen N Luteran M Pillai S NK T cells provide lipid antigen-specific cognate help for B cells. Proc Natl Acad Sci USA (2008) 105:8339–44. 10.1073/pnas.0801375105 18550809 PMC2448838

[43] O’Reilly V Zeng S Bricard G Atzberger A Hogan AE Jackson J Distinct and overlapping effector functions of expanded human CD4+, CD8α+ and CD4-CD8α- invariant natural killer T cells. PLoS One (2011) 6:e28648. 10.1371/journal.pone.0028648 22174854 PMC3236218

[44] Zeng SG Ghnewa YG O’Reilly VP Lyons VG Atzberger A Hogan AE Human invariant NKT cell subsets differentially promote differentiation, antibody production and T cell stimulation by B cells in vitro. J Immunol (2013) 191:1666–76. 10.4049/jimmunol.1202223 23851681 PMC4201948

[45] Leslie DS Vincent MS Spada FM Das H Sugita M Morita CT CD1-mediated γ/δ T cell maturation of dendritic cells. J Exp Med (2002) 196:1575–84. 10.1084/jem.20021515 12486100 PMC2196072

[46] Collins C Wolfe J Roessner K Shi C Sigal LH Budd RC. Lyme arthritis synovial γδ T cells instruct dendritic cells via fas ligand. J Immunol (2005) 175:5656–65. 10.4049/jimmunol.175.9.5656 16237055

[47] Mangan BA Dunne MR O’Reilly VP Dunne PJ Exley MA Doherty DG. Cutting edge: CD1d restriction and Th1/Th2/Th17 cytokine secretion by human Vδ3 T cells. J Immunol (2013) 191:30–4. 10.4049/jimmunol.1300121 23740951 PMC3721026

[48] Russano AM Agea E Corazzi L Postle AD De Libero G Porcelli S Recognition of pollen-derived phosphatidyl-ethanolamine by human CD1d-restricted γδ T cells. J Allergy Clin Immunol (2006) 117:1178–84. 10.1016/j.jaci.2006.01.001 16675349

[49] Meraviglia S Eberl M Vermijlen D Todaro M Buccheri S Cicero G In vivo manipulation of Vγ9Vδ2 T cells with zoledronate and low-dose interleukin-2 for immunotherapy of advanced breast cancer patients. Clin Exp Immunol (2010) 161:290–7. 10.1111/j.1365-2249.2010.04167.x 20491785 PMC2909411

[50] Kalyan S Wesch D Kabelitz D. Aminobisphosphonates and Toll-like receptor ligands: recruiting Vγ9Vδ2 T cells for the treatment of hematologic malignancy. Curr Med Chem (2011) 18:5206–16. 10.2174/092986711798184280 22087821

[51] Santolaria T Robard M Léger A Catros V Bonneville M Scotet E. Repeated systemic administrations of both aminobisphosphonates and human Vγ9Vδ2 T cells efficiently control tumor development in vivo. J Immunol (2013) 191:1993–2000. 10.4049/jimmunol.1300255 23836057

