# Supplementary material for: Human Vδ2+ γδ T Cells Differentially Induce Maturation, Cytokine Production, and Alloreactive T Cell Stimulation by Dendritic Cells and B Cells
Source: Front Immunol. 2014 Dec 19;5:650. doi: 10.3389/fimmu.2014.00650 (PMC4271703; doi:10.3389/fimmu.2014.00650)
Supplement: Supplementary file 1 [file Presentation_1.PDF]

Supplementary Material

Human Vδ2[+] γδ T cells differentially induce maturation, cytokine production and alloreactive T cell stimulation by dendritic cells and B cells

Andreea Petrasca[1], Derek G. Doherty[2]*

[1]Division of Immunology, School of Medicine, Trinity College Dublin, University of Dublin, Ireland

*** Correspondence:** Derek Doherty, Discipline of Immunology, School of Medicine, Trinity College Dublin, Ireland.
derek.doherty@tcd.ie

Supplementary Figures

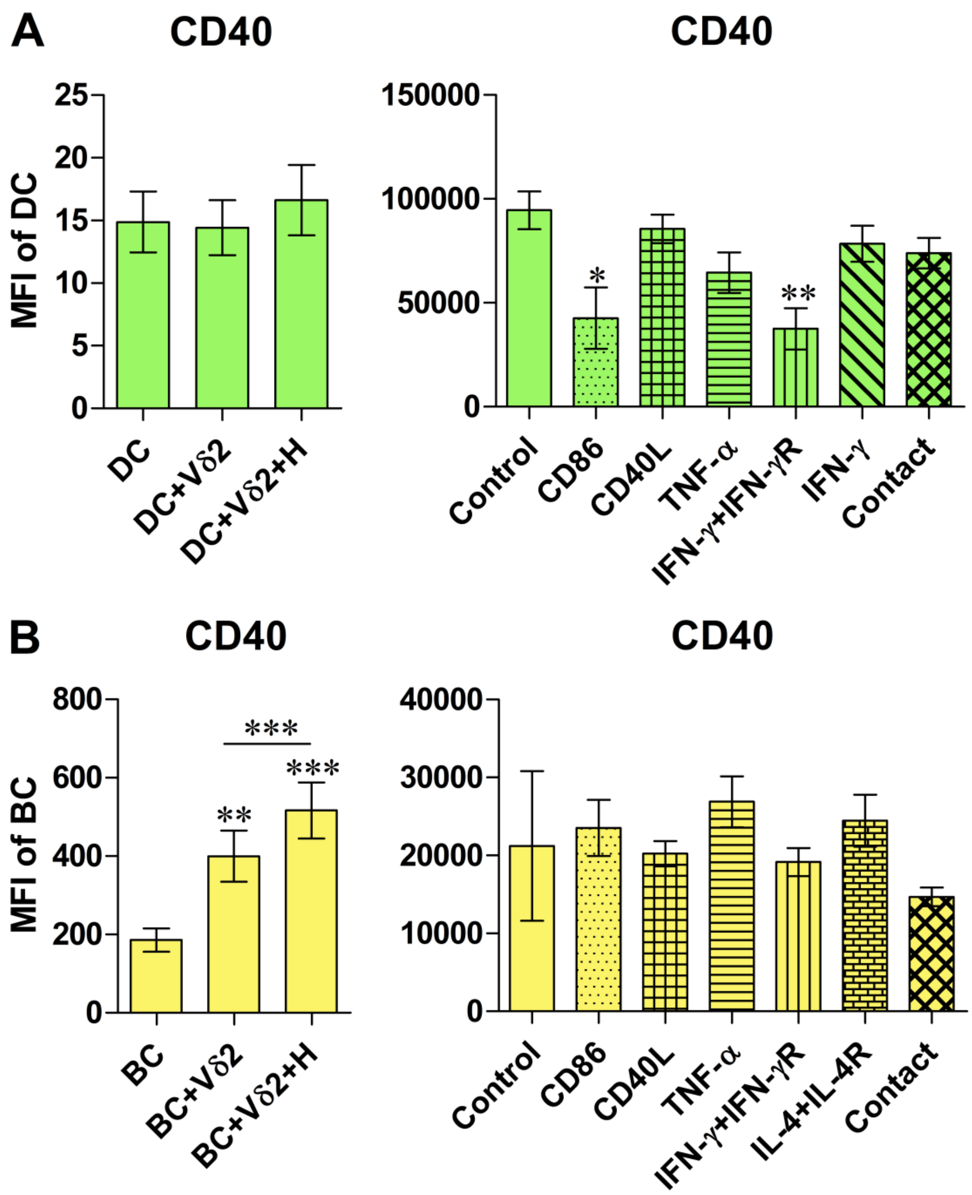

Supplementary Figure 1. Vδ2 T cells induce the expression of CD40 by B cells (BC). Monocyte-derived DC or enriched peripheral blood B cells were co-cultured for 24 h or 72 h with HMB-PP-expanded human Vδ2 T cells in the absence or presence of HMB-PP (denoted H). Cells were then stained using mAbs specific for CD11c (DC) or CD19 (BC) and CD40 and analyzed by flow cytometry. Left panels show average (±SEM) mean fluorescence intensities (MFI) of staining for CD40 expression by (A) DC (n=7) and (B) B cells (n=7). Right panels show average (±SEM) MFI of staining for CD40 by DC or B cells after co-culturing them with Vδ2 T cells in the absence (control) or presence of blocking mAbs specific for CD86, CD40L, TNF-α, IFN-γ + IFN-γR, IL-4 + IL-4R or with the DC or B cells separated from Vδ2 T cells using transwell inserts (n=5 for DC treatments and n=3 for BC treatments). *p<0.05, **p<0.01, ***p<0.001 using a paired *t* test compared to DC or BC alone (left panels) or compared to BC control (right panels) and unpaired *t* test compared to DC control (right panels) except where indicated by horizontal lines.

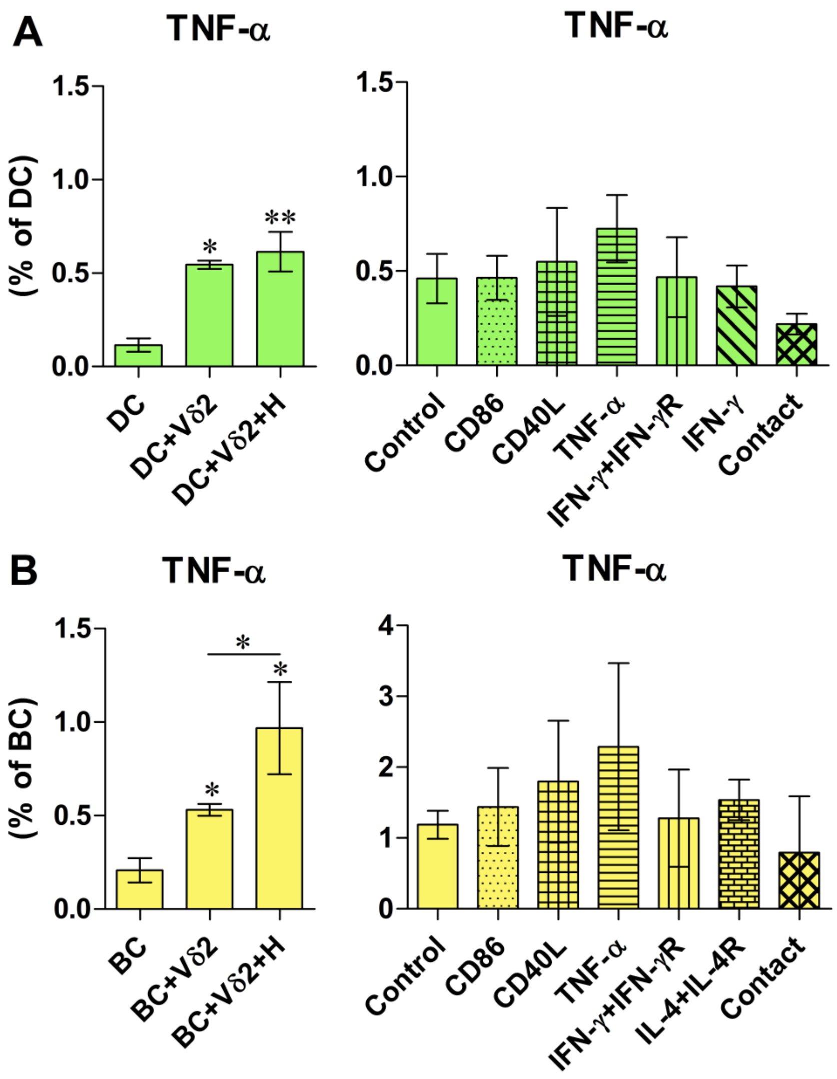

Supplementary Figure 2. Vδ2 T cells induce low amounts of TNF-α expression by DC and B cells. DC or B cells were co-cultured with HMB-PP-expanded human Vδ2 T cells in the absence or presence of HMB-PP (denoted H) for 24 h. The cultures were then treated with monensin for a further 16 h and stained for cell surface expression of CD11c or CD19 and intracellular expression of TNF-α by gated CD11c+ cells (DC) and $CD19^+$ cells (BC), respectively. Left panels show mean (±SEM) percentages of (A) DC (n=3-9) and (B) BC (n=3-6) expressing TNF-α. Right panels show mean (±SEM) percentages of (A) DC and (B) BC expressing TNF-α respectively, after co-culturing them with Vδ2 T cells in the absence (control) or presence of blocking mAbs specific for CD86, CD40L, TNF-α, IFN-γ + IFN-γR, IL-4 + IL-4R or with the DC or B cells separated from Vδ2 T cells using transwell inserts (n=5 for DC treatments and n=3 for BC treatments). *p<0.05, **p<0.01, using a paired t test compared to DC or BC alone (left panels) or compared to BC control (right panels) and unpaired t test compared to DC control (right panels) except where indicated by horizontal lines.

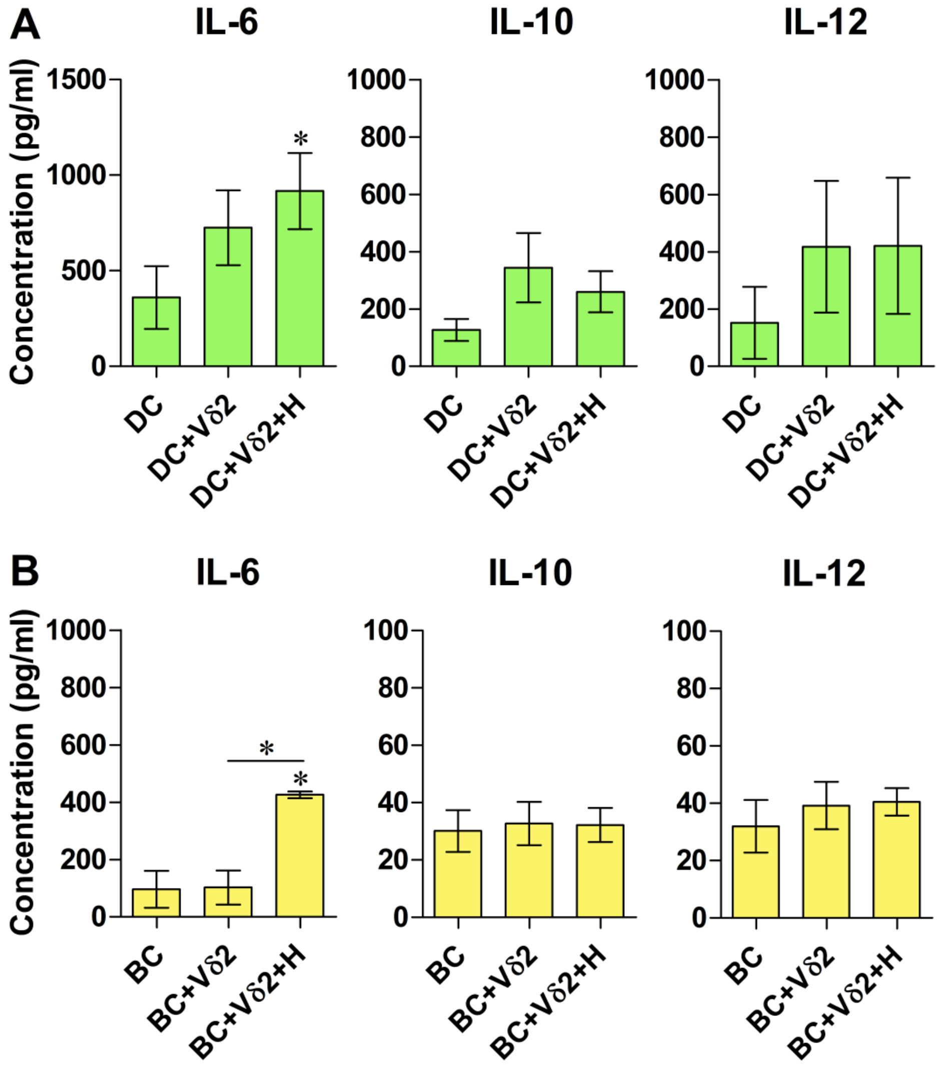

Supplementary Figure 3. Vδ2 T cells induce IL-6, but not IL-10 or IL-12 secretion by DC and B cells. DC or B cells were co-cultured with HMB-PP-expanded human Vδ2 T cells in the absence or presence of HMB-PP (denoted H). After 24 h (for DC) or 72 h (for BC), supernatants were harvested and analyzed for IL-6, IL-10 and IL-12p70 by ELISA. Results show mean (±SEM) concentration of IL-6, IL-10 and IL-12p70 from the (A) DC (n=4-11) and (B) BC (n=3-7) co-cultures. *$p<0.05$ using a paired *t* test compared to DC or BC alone except where indicated by horizontal lines.

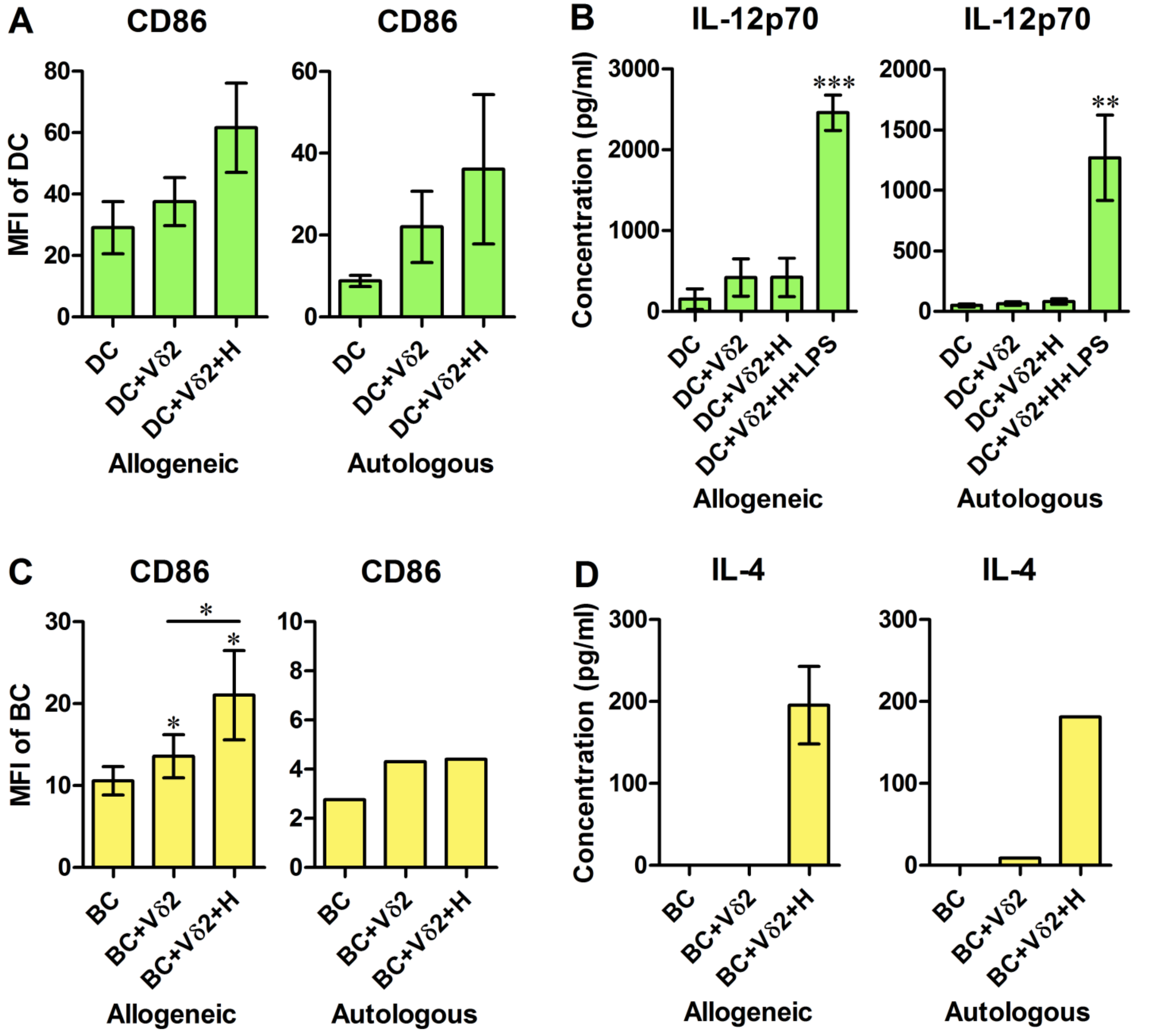

Supplementary Figure 4. Allogeneic and autologous Vδ2 T cells equally activate DC and B cells. DC or B cells (BC) were co-cultured with autologous or allogeneic HMB-PP-expanded human Vδ2 T cells in the absence or presence of HMB-PP (denoted H) or LPS. After 24 h (for DC) or 72 h (for BC), the cells were stained with mAbs for CD11c (DC) or CD19 (BC) and CD86 and analyzed by flow cytometry. The supernatants were harvested and examined for IL-4 or IL-12p70 secretion by ELISA. Results show average (±SEM) mean fluorescence intensities of staining for CD86 expression by (A) DC (n=3-8) or (C) B cells (n=1-12) for allogeneic (left panels) and autologous (right panels) co-cultures. Results show mean (±SEM) concentration of (B) IL-12p70 (n=10-11) concentration from DC co-cultures and (D) IL-4 (n=1-2) from BC co-cultures for allogeneic and autologous co-cultures. *p<0.05, **p<0.01, ***p<0.001 using a paired t test compared to DC or BC alone except where indicated by horizontal lines.

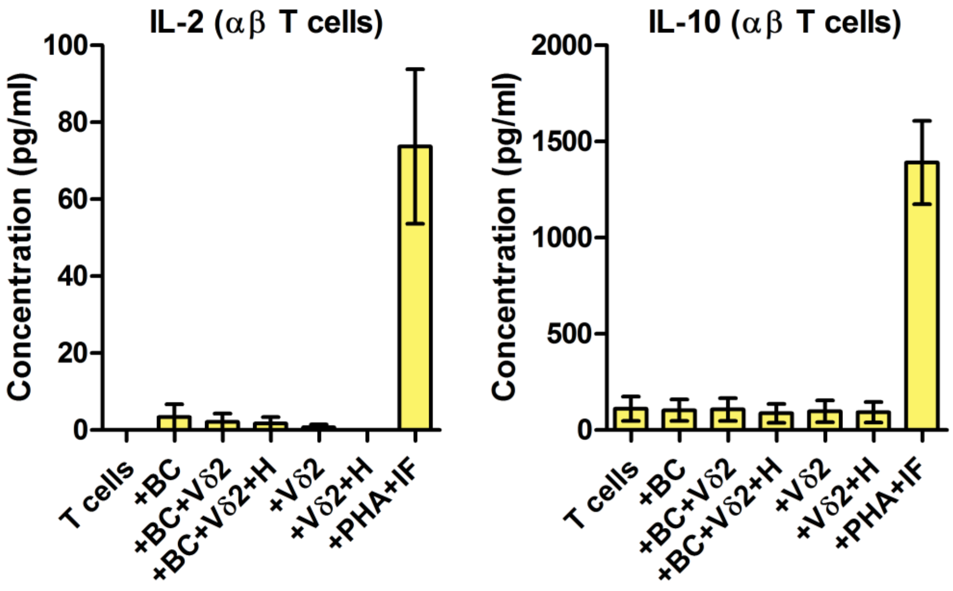

Supplementary Figure 5. Vδ2 T cell-matured B cells do not induce IL-2 or IL-10 production by alloreactive T cells. B cells were co-cultured with HMB-PP-expanded human Vδ2 T cells in the absence or presence of HMB-PP (denoted H). After 24 h, resting αβ T cells were added to the culture at a ratio of 10:1. A positive control of CD3 T cells treated with PHA-P and irradiated feeders (denoted IF) was set up. After a further 3 days, supernatants were harvested and analyzed for IL-2 and IL-10 levels by ELISA. Results show mean (±SEM) concentration of IL-2 and IL-10 from the co-cultures (n=4).
